# How Does Replacement of the Axial Histidine Ligand in Cytochrome *c* Peroxidase by N_δ_-Methyl Histidine Affect Its Properties and Functions? A Computational Study

**DOI:** 10.3390/ijms21197133

**Published:** 2020-09-27

**Authors:** Calvin W. Z. Lee, M. Qadri E. Mubarak, Anthony P. Green, Sam P. de Visser

**Affiliations:** 1 Manchester Institute of Biotechnology, The University of Manchester, 131 Princess Street, Manchester M1 7DN, UK; calvin.wz.lee@gmail.com (C.W.Z.L.); qadri.fendy@gmail.com (M.Q.E.M.); anthony.green@manchester.ac.uk (A.P.G.); 2 Department of Chemical Engineering and Analytical Science, The University of Manchester, Oxford Road, Manchester M13 9PL, UK; 3 Department of Chemistry, The University of Manchester, Oxford Road, Manchester M13 9PL, UK

**Keywords:** density functional theory, enzyme models, epoxidation, hydroxylation, heme enzymes, peroxidases, enzyme engineering

## Abstract

Heme peroxidases have important functions in nature related to the detoxification of H_2_O_2_. They generally undergo a catalytic cycle where, in the first stage, the iron(III)–heme–H_2_O_2_ complex is converted into an iron(IV)–oxo–heme cation radical species called Compound I. Cytochrome *c* peroxidase Compound I has a unique electronic configuration among heme enzymes where a metal-based biradical is coupled to a protein radical on a nearby Trp residue. Recent work using the engineered N_δ_-methyl histidine-ligated cytochrome *c* peroxidase highlighted changes in spectroscopic and catalytic properties upon axial ligand substitution. To understand the axial ligand effect on structure and reactivity of peroxidases and their axially N_δ_-methyl histidine engineered forms, we did a computational study. We created active site cluster models of various sizes as mimics of horseradish peroxidase and cytochrome *c* peroxidase Compound I. Subsequently, we performed density functional theory studies on the structure and reactivity of these complexes with a model substrate (styrene). Thus, the work shows that the N_δ_-methyl histidine group has little effect on the electronic configuration and structure of Compound I and little changes in bond lengths and the same orbital occupation is obtained. However, the N_δ_-methyl histidine modification impacts electron transfer processes due to a change in the reduction potential and thereby influences reactivity patterns for oxygen atom transfer. As such, the substitution of the axial histidine by N_δ_-methyl histidine in peroxidases slows down oxygen atom transfer to substrates and makes Compound I a weaker oxidant. These studies are in line with experimental work on N_δ_-methyl histidine-ligated cytochrome *c* peroxidases and highlight how the hydrogen bonding network in the second coordination sphere has a major impact on the function and properties of the enzyme.

## 1. Introduction

Heme peroxidases perform a wide range of functions in biology, ranging from defense mechanisms against infection to hormone biosynthesis in the human body [1,2,3,4,5,6,7,8,9]. The general reaction catalyzed by peroxidases is the reduction, and hence detoxification, of H_2_O_2_ to two water molecules, see Equation (1), which requires two external protons and electrons. In heme peroxidases, this reaction takes place by the binding of H_2_O_2_ to the distal site of the heme, while intricate proton and electron transfer pathways channel protons and electrons to and from the heme. In general, the peroxidases undergo a catalytic cycle starting from a resting state, i.e., a water-bound heme group, where the water ligand is substituted by H_2_O_2_. This H_2_O_2_ binding triggers a proton relay that converts the H_2_O_2_-bound heme into water and an iron(IV)–oxo heme cation radical intermediate, called Compound I (Cpd I). Structurally, the heme iron atom is bound to the protein through a histidine linkage in the axial position that connects the heme to the protein [10,11,12,13,14]. In contrast to the peroxidases, the heme mono-oxygenases, such as the cytochromes P450, bind the heme through a cysteinate ligand [1,2,15,16,17,18,19,20,21,22,23,24]. The difference in the protein binding pattern between the peroxidases and mono-oxygenases generally has an effect on the properties of the iron/heme co-factor as the more electron donating cysteinate ligand induces a stronger “push” effect compared with the neutral histidine-ligated system [25,26,27]. As a consequence, the peroxidases have different redox properties over the mono-oxygenases, which affects their (bio)chemical functions. Indeed, mutations of the axial ligand or its direct environment affect the structure and catalytic ability of the protein [28,29,30,31].

H_2_O_2_ + 2H^+^ + 2e^−^ → 2 H_2_O (1)

In recent years, efforts have been made to engineer proteins to study their chemical functions and properties and find applications in biotechnology [32,33,34,35,36]. For instance, the nonheme iron dioxygenase *S*-mandalate synthase was engineered into *R*-mandalate synthase through substrate-binding pocket modifications that positioned the substrate differently [36]. Several attempts have been reported in the literature on the engineering of peroxidases with the aim to change their chemical properties and reactivity patterns [37,38]. In particular, first- and second-coordination sphere effects around the heme co-factor can lead to dramatic changes in the electronic properties of the metal center and consequently its conversion of substrates into products [39].

**Figure 1:**
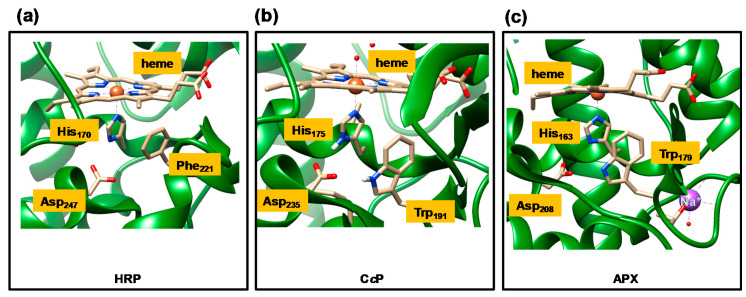
Active site structures of (**a**) horseradish peroxidase (HRP; 1H58 pdb), (**b**) cytochrome *c* peroxidase (C*c*P; 1CCA pdb), (**c**) ascorbate peroxidase (APX; 1OAF pdb).

To understand the axial ligand differences in peroxidases, we show three typical structures in Figure 1: one with an axial hydrogen-bonded diad, one with an axial hydrogen-bonded triad and one with a nearby cation-binding site. Figure 1 displays the active site structures of horseradish peroxidase (HRP) [40], cytochrome *c* peroxidase (C*c*P) [41] and ascorbate peroxidase (APX) [42], as taken from the protein databank (pdb) [43]. At first sight, they look very similar and all contain an iron(heme) linked to the protein through a proximal histidine residue: His_170_ in HRP, His_175_ in C*c*P and His_163_ in APX. In all three proteins, the proximal (also called axial) histidine ligand is part of a hydrogen bonding network and interacts with the carboxylate of an Asp residue (Asp_247_ in HRP, Asp_235_ in C*c*P and Asp_208_ in APX), which imparts a degree of imidazolate-like character onto the axial ligand, thus increasing its electron-donating capabilities towards the heme. The proximal hydrogen-bonding network is further extended in C*c*P and APX where the Asp carboxylate also forms a hydrogen bond with a proton from a nearby indole group of a Trp residue (Trp_191_ in C*c*P and Trp_179_ in APX). Interestingly, this residue is absent in HRP, where the Phe_221_ group is seen in that position. An additional difference in APX is the presence of a cation (Na^+^) binding site nearby the Trp_179_ residue, although its function is not exactly clear.

**Scheme 1:**
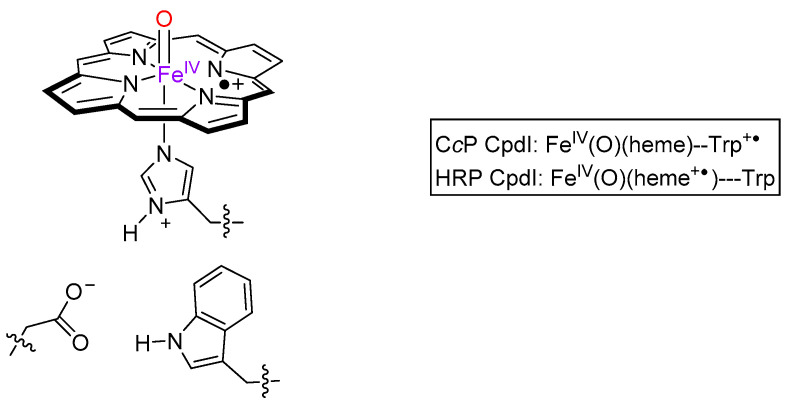
Chemical structure and electronic configuration of Compound I of peroxidases.

Despite their differences in structural features, the three peroxidases undergo the same catalytic mechanism that binds and utilizes H_2_O_2_ on the distal site of the heme and react it to two water molecules via the formation of an iron(IV)–oxo heme cation radical called Compound I (Cpd I) intermediate. A proton shuttle mechanism on the distal site converts H_2_O_2_ to Cpd I [44,45,46,47]. Experimentally, Cpd I of C*c*P has been well characterized with electron paramagnetic resonance and UV-Vis spectroscopic methods and found to be a key intermediate in the catalytic cycle [48,49,50,51,52]. However, the Cpd I species in C*c*P, APX and HRP show differences in electronic configuration that have been assigned as resulting from the structural differences in the proximal pocket and, in particular, the proximal hydrogen bonding network. In particular, C*c*P Cpd I has a protein radical (on Trp_191_), while HRP and APX have a heme-based radical. Extensive computational studies using density functional theory, quantum mechanics/molecular mechanics and molecular dynamics studies have been performed on the various stages of the catalytic cycle of peroxidases and particularly on Cpd I [44,45,46,47,53,54,55,56,57,58,59,60]. These studies (Scheme 1) confirmed Cpd I as a triradical species with three unpaired electrons, whereby two of those are on the metal (in the π*_xz_ and π*_yz_ orbitals), while the third radical is non-metal based [53,57,61]. Thus, in P450 Cpd I and HRP Cpd I, the third unpaired electron is typically based in a heme orbital with a_2u_ symmetry [44,53,55,62,63,64,65,66,67,68,69,70]. By contrast, C*c*P displays a protein radical that is located on a nearby Trp residue (Trp_191_). Interestingly, even though APX also has this hydrogen-bonding axial triad, its Cpd I structure does not display a Trp radical, but it remains on the heme instead. These differences in electronic configuration were explained through the presence of a cation binding site of a Na^+^ ion that induced long-range electrostatic interactions. Thus, engineering of the cation binding site in C*c*P changed the electronic properties and stabilized a heme radical over a Trp radical in Cpd I [71]. Gas-phase density functional theory calculations [72] on C*c*P Cpd I, however, gave mixed radical character delocalized over both the heme and the Trp groups. The addition of point charges to the model in the position of the cation binding site polarized the charges better and either gave a pure Trp radical or a heme radical dependent on the sign of the point charge [57,73]. As such, the local environment is important for the correct description of the electronic configuration of Cpd I of peroxidases.

More recent studies [37,38] on engineered forms of APX and myoglobin where the axial histidine ligand is replaced by N_δ_-methyl histidine showed major changes in terms of redox properties and catalysis. Thus, the incorporation of N_δ_-methyl histidine (HisMe) as axial ligand breaks the hydrogen bonding network on the axial site of the heme. In particular, it breaks the hydrogen bonding interaction between the axial ligand and Asp_208_ groups and consequently changes the heme properties. Interestingly, the turnover numbers of guaiacol oxidation were enhanced by a factor of five in the engineered APX. Similar work on the oxygen binding protein myoglobin, which lacks the hydrogen bonded axial triad of natural peroxidases, was also carried out. Interestingly, this study found that the introduction of the non-canonical HisMe ligand led to a substantial increase in the k_cat_ value of guaiacol oxidation. More recently, a genetically encoded HisMe ligand in C*c*P was explored and studies investigated the change in axial ligand electron donation and ferryl heme reactivity.

These studies clearly demonstrate the replacement of a histidine group (His) by N_δ_-methyl histidine has an effect on the structure, electronic properties and reactivity of heme peroxidases, and in some cases can lead to biocatalysts with augmented properties. As experimental studies failed to give a detailed electronic insight into the effects of these minor changes to the protein structure and the reactivity properties of the oxidant, we decided to do a detailed computational study. To gain an insight into the effects that influence the active site, we elected to perform a detailed density functional theory (DFT) study on models mimicking the various peroxidases and their engineered forms with the axial histidine replaced by N_δ_-methyl histidine. The aims of our studies are to gain an insight into how N-methylation of an axial histidine ligand in heme peroxidases affects the electronic configuration, chemical properties and reactivity patterns.

## 2. Results

**Scheme 2:**
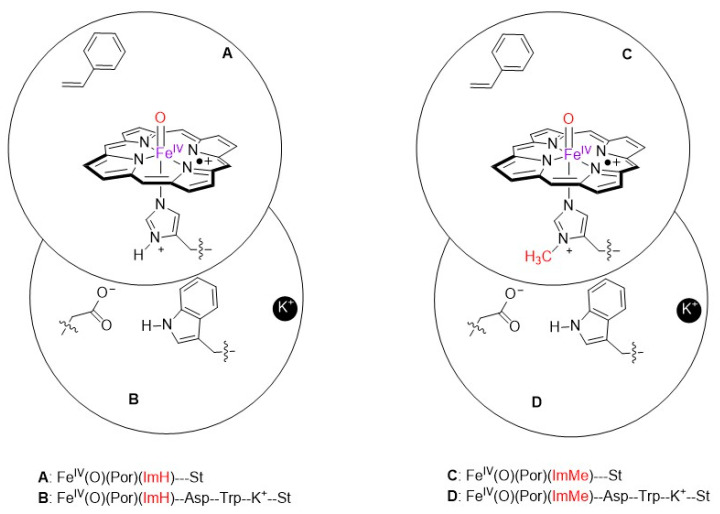
Models **A**, **B**, **C** and **D**, studied as models of peroxidases and N_δ_-methyl histidine (HisMe)-engineered peroxidases, as investigated in this work. Por stands for porphyrin, St is styrene, ImH is imidazole and ImMe is methyl imidazole.

To gain an insight into the structure and reactivity of N_δ_-methyl histidine-ligated peroxidases, we created active site models of HRP and C*c*P for wild-type and engineered structures. The Cpd I models were built from available crystal structure coordinates by inserting an oxo group in the distal ligand position. HRP Cpd I was modelled as a minimal model with the heme group approximated as protoporphyrin IX, with all side chains replaced by hydrogen atoms, an oxo in the distal position and with imidazole as an axial ligand bound to iron (model **A**, Scheme 2). Previous calculations showed that the heme periphery substituents pay little contribution to the electronic configuration of Cpd I and hence were shorted to hydrogen atoms in our models [74,75,76,77]. The C*c*P model used the first-coordination sphere of the HRP model **A** and was expanded with the axial residues that form the hydrogen-bonded network around the His ligand, namely an acetate group (mimicking Asp_235_) and an indole group (representing Trp_191_) to get model **B**. In previous work [72] it was shown that this model gives a mixed porphyrin-Trp radical in Cpd I and hence is a poor mimic of C*c*P Cpd I. However, it was shown that the inclusion of a point-charge in the model polarized the charge distributions better and gave either a pure Trp radical (with positive charge) or a pure porphyrin radical (with negative charge) [73]. Therefore, model **B** included a nearby K^+^ ion to obtain an electronic configuration for Cpd I that reflects the experimental electron paramagnetic resonance (EPR) data for C*c*P. The positions of the methyl carbons of the Asp_235_ and Trp_191_ and the K^+^ ion were fixed with respect to the iron atom to keep the structure as close as possible to the crystal structure coordinates. Subsequently, we engineered the axial imidazole ligand of the HRP and C*c*P models, i.e., models **A** and **B**, to N_δ_-methyl imidazole to get the HisMe engineered models of HRP and C*c*P: models **C** and **D**. All models have an overall charge of +1 and were calculated in the lowest energy doublet and quartet spin states as identified with a superscript. We studied the electronic configuration and physicochemical properties of these Cpd I models as well as the reactivity with a model substrate, i.e., styrene, which is a commonly used substrate for oxygen atom transfer reactions [78,79]. 

Let us first look into the structural and electronic differences between the four model complexes. In the smallest model that only considers the iron(IV)–oxo porphyrin cation radical with either imidazole (model **A**) or N_δ_-methyl imidazole (model **C**) as the axial ligand, we get almost identical structures. Thus, the Fe–O distances obtained for ^2^**A**, ^4^**A**, ^2^**C** and ^4^**C** are 1.663, 1.664, 1.664 and 1.665 Å, respectively, while the proximal Fe–N bond distances are 2.083, 2.079, 2.081 and 2.076 Å for the same set of structures. As such, the replacement of imidazole by N_δ_-methyl imidazole has little effect on the optimized geometries of the minimal models mimicking HRP. In all cases, an electronic configuration of π*_xz_^1^ π*_yz_^1^ a_2u_^1^ is obtained, whereby the two π* orbitals are up-spin and the a_2u_ orbital is either up-spin or down-spin in the quartet or doublet spin state. The π*_xz_ and π*_yz_ orbitals represent the antibonding interaction between the metal 3d_xz_/3d_yz_ orbitals with a 2p orbital on the oxygen atom. The a_2u_ orbital is a heme-type π* orbital, which in D_4h_ symmetry has the a_2u_ label. As such, the structure and electronic configuration between the two HRP models is the same. Consequently, structures **A** and **C** give a spin density of about one on the Fe, O and Por groups. Indeed, the spin density values for structures **A** and **C** are within 0.01 unit of each other. This is not surprising as the valence orbitals show little contribution of the axial histidine ligand. Consequently, the replacement of imidazole by N_δ_-methyl imidazole has little electronic effect on the small active site model of HRP and minimal changes to the first-coordination sphere of histidine-ligated heme complexes.

**Figure 2:**
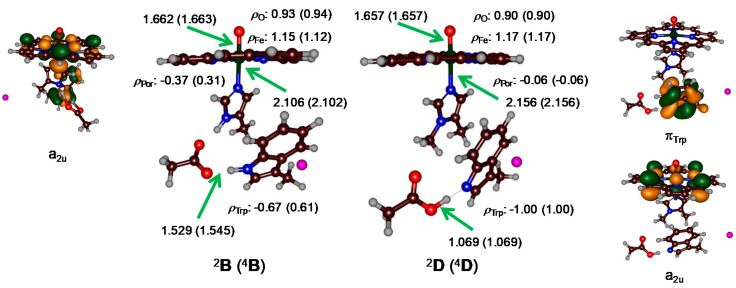
Optimized geometries of Compound I of C*c*P and N_δ_-methyl histidine engineered C*c*P as obtained with models **B** and **D**. Bond lengths are in angstroms and group spin densities in au. Singly occupied protein orbitals are given on the side. Quartet spin data in parenthesis.

Of course, models **A** and **C** lack the axial hydrogen bonding network and, particularly, the accepting hydrogen bond from the Asp carboxylic acid. To gain an insight into how the cleavage of these axial hydrogen bonding interactions affects the structure and charge distributions, we analyzed the differences in the properties and reactivity of histidine and N_δ_-methyl histidine-ligated C*c*P models (structures ^2,4^**B** and ^2,4^**D**). Figure 2 displays the optimized geometries and group spin densities of ^2,4^**B** and ^2,4^**D**. As can be seen, small but significant changes in geometry are obtained between ^2,4^**B** on the one hand and ^2,4^**D** on the other hand. Thus, the Fe–O distance shortens from 1.662 to 1.657 Å between ^2^**B** and ^2^**D**, while the Fe–N(His) distance elongates from 2.106 to 2.156 Å. Note that during the geometry optimization of ^2,4^**D**, the indole proton moved closer to the carboxylate of Asp_235_ in the N_δ_-methyl histidine engineered systems. Therefore, the removal of the His–Asp hydrogen bond in engineered model **D** changes the local pK_a_ of the carboxylate group and makes it more acidic. This may be the reason for the slightly more radical character of the Trp unit in the N_δ_-methyl histidine-engineered structures.

Electronically, all **B** and **D** model species are described by the same molecular orbital occupation with unpaired electrons as π*_xz_, π*_yz_ and π_Trp_ orbitals. The former two are the antibonding interactions along the Fe–O axis, whereas the π_Trp_ orbital is a π-orbital on the axial Trp residue. In addition, the high-lying porphyrin orbital, i.e., a_2u_, is doubly occupied in ^2,4^**B** and ^2,4^**D**. As such structures, **B** and **D** can be described with the overall electronic configuration π*_xz_^1^ π*_yz_^1^ a_2u_^2^ π_Trp_^1^, whereby the doublet spin states have the Trp radical as a down-spin, while it is an up-spin in the quartet spin states. Thus, the addition of the hydrogen-bonding distal network and the point charge to the minimal models (**A**/**C**) to get **B**/**D** leads to the polarization of the charge distributions and gives a Trp radical rather than a heme radical. This is in agreement with experimental electron paramagnetic resonance (EPR) and electron nuclear double resonance (ENDOR) spectroscopy studies that located a Trp radical in C*c*P Cpd I [48,49].

There are differences between structures **B** and **D**; however, these are particularly related to the π_Trp_ orbital and, therefore, Figure 2 displays the singly occupied a_2u_ and π_Trp_ orbitals of ^2^**B** and ^2^**D**. In ^2,4^**B**, the π_Trp_ orbital mixes somewhat with a porphyrin a_2u_-type orbital and, as a result, partial spin density on the porphyrin scaffold is obtained (0.31 in ^4^**B** and −0.37 in ^2^**B**) and on the Trp residue (0.61 in ^4^**B** and −0.67 in ^2^**B**). Indeed, the two orbitals are either localized on the porphyrin (a_2u_) or the Trp unit (π_Trp_). As a result, in structure **D**, all spin density associated with the single occupation of the π_Trp_ orbital is located on the Trp unit and little is located on the porphyrin manifold.

**Scheme 3:**
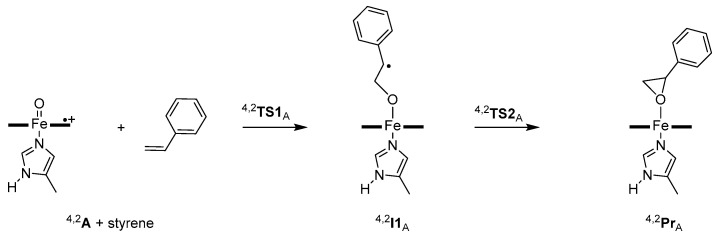
Reaction mechanism with labeling of the individual structures.

To test the catalytic effects of engineering the axial histidine ligand in peroxidases to N_δ_-methyl histidine, we investigated the mechanism of oxygen atom transfer from Cpd I to a substrate with styrene as a model substrate. Thus, styrene epoxidation is a typical model reaction for double bond activation and styrene has been used as model substrate in many previous studies for biomimetic and enzymatic reaction mechanisms [78,79,80,81,82]. The styrene epoxidation was studied by the minimal heme models ^2,4^**A** and ^2,4^**C**, which contain the iron(IV)–oxo group inside a bare porphyrin and with imidazole or N_δ_-methyl imidazole as the axial ligand. These minimal models should give an insight into the electrostatic effects of replacing the axial ligand with N_δ_-methyl histidine on the styrene epoxidation reaction. Subsequently, the same reaction was also explored for the expanded models ^2,4^**B** and ^2,4^**D** that show the effect of the proximal hydrogen bonding network and the second-coordination sphere on structure and reactivity. The overall reaction mechanism explored for all systems (^4,2^**A**, ^4,2^**B**, ^4,2^**C** and ^4,2^**D**), although only the general pathway, is shown in Scheme 3 for model **A**. The same labels of the transition states and local minima are used for the reactions for model **B**, **C**, and **D** and these letters are given in subscript after the label. The reactions start from a long-range complex of styrene with the Cpd I model, i.e., an iron(IV)–oxo heme cation radical, for models **A**, **B**, **C**, and **D** in the doublet and quartet spin states. Subsequently, we follow the reaction for the C–O bond formation with the terminal carbon atom of styrene to form a radical intermediate (**I1**) via a C–O bond formation transition state **TS1**. Thereafter, a ring-closure transition state (**TS2**) separates the radical intermediates from epoxide product complexes (**Pr**). This mechanism was explored previously for double bond epoxidation by Cpd I of P450 and peroxidase model complexes [76,77,78,79,80,81,82,83,84,85,86,87,88,89,90,91,92] as well as engineered peroxidase systems with modified axial ligands [93,94]. In general, in these studies, **TS1** is the rate-determining step and **TS2** is small or negligible, and the overall reaction is highly exothermic.

**Figure 3:**
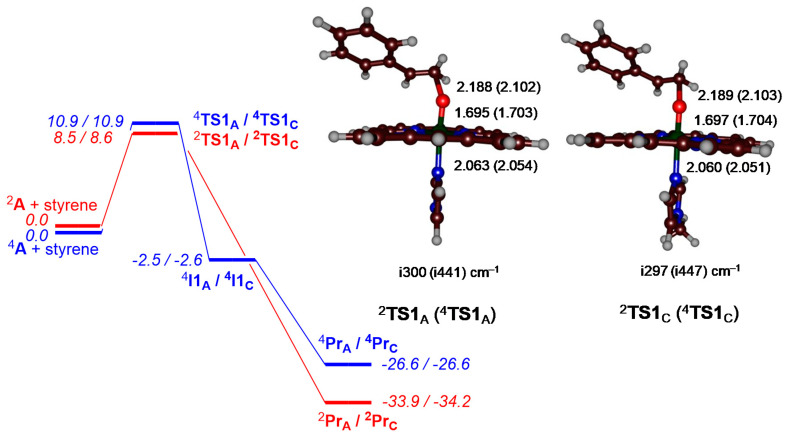
Potential energy landscape of styrene epoxidation by ^2,4^**A** and ^2,4^**C** as calculated at UB3LYP/BS1 in Gaussian-09. Energies (UB3LYP/BS1//UB3LYP/BS2+ZPE+E_solv_) are in kcal mol^−1^ with respect to isolated reactants and give values for the reaction of **A**/**C** and doublet spin results in red and quartet spin results in blue. Optimized geometries of the rate-determining transition states give bond lengths in angstroms and the imaginary frequency in cm^−1^.

Next, we studied the epoxidation pathways for models **A**, **B**, **C** and **D** and attempted to locate radical intermediates, products and the transition states that separate these local minima. Let us start with analyzing the pathways for the small model complexes **A** and **C**; the reaction mechanisms for those models are given in Figure 3. The reactions start with an initial C–O bond formation transition state (**TS1**_A_, **TS1**_C_). In the low-spin pathways beyond the C–O bond formation transition state, the system relaxes to epoxide product complexes (^2^**Pr**_A_, ^2^**Pr**_C_) directly without the formation of a radical intermediate. Attempts to characterize these radical intermediates failed and the geometry optimization fell to the product complexes directly. In the high-spin pathways, by contrast, a small shoulder is found with a local minimum for a radical intermediate (^4^**I1**_A_, ^4^**I1**_C_). However, no ring-closure transition state could be identified and the constraint geometry scans show a fast conversion to epoxide products (^4^**Pr**_A_, ^4^**Pr**_C_) with a negligible ring-closure barrier. Therefore, both doublet and quartet spin pathways can be considered as concerted reaction mechanisms that proceed via a rate-determining C–O bond formation barrier. Previous work on the epoxidation by Cpd I porphyrin systems also found a rate-determining C–O bond formation leading to a radical intermediate with two-state reactivity patterns in competing doublet and quartet spin states [76,78,79,80,81,82,83,84,85,86,87,88,89,90,95]. Interestingly, calculations on a pentacoordinated Cpd I model without an axial ligand gave epoxidation pathways similar to those in Figure 3 with a rate-determining C–O bond formation followed by a fast and highly exothermic process to epoxide products [76]. Therefore, the lack of a stable radical intermediate here may have to do with the binding of a weak (neutral) axial ligand. With an anionic ligand in the axial position, more charge density is donated to the iron(IV)–oxo and, on top of that, the metal is pulled into the plane of the heme, which stabilizes the radical intermediate. The same is seen in aliphatic hydroxylation reactions, where the reaction is generally stepwise with an initial hydrogen atom abstraction followed by OH rebound. Strong electron-donating ligands affect the first electron transfer and stabilize the radical intermediate [95,96,97,98]. The obtained epoxidation barriers, calculated at UB3LYP/BS2//UB3LYP/BS1+ZPE+E_solv_ with ZPE = zero-point energy and E_solv_ the solvation energy, are shown in Figure 3. The doublet spin barriers are the lowest at 8.5 kcal mol^−1^ for model **A** and 8.6 kcal mol^−1^ for model **C**. The quartet spin barriers are slightly higher and values of 10.9 kcal mol^−1^ are calculated for both model **A** and **C**. Therefore, the replacement of the axial imidazole ligand by N_δ_-methyl imidazole has a negligible effect on the chemical properties of the oxidant and gives the same structures and epoxidation barrier heights.

Optimized geometries of ^4,2^**TS1**_A_ and ^4,2^**TS1**_C_ are given on the right-hand side of Figure 3. The structures look similar to previously reported epoxidation transition states for styrene by Cpd I models [76,78,79,80,81,82]. The pair of doublet spin transition states are very much alike and so is the pair of quartet spin structures. Thus, the substrate approaches the oxo group sideways and elongates the Fe–O distance to 1.695 (1.697) Å in ^2^**TS1**_A_ (^2^**TS1**_C_) and 1.703 (1.704) Å in ^4^**TS1**_A_ (^4^**TS1**_C_). At the same time, the O–C distance shortens to 2.188 (2.189) Å in ^2^**TS1**_A_ (^2^**TS1**_C_) and 2.102 (2.103) Å in ^4^**TS1**_A_ (^4^**TS1**_C_). The imaginary frequencies represent a C–O stretch vibration and their magnitude varies from i297 to i447 cm^−1^, which is typical for epoxidation transition states [79]. Nevertheless, the imaginary mode confirms these transition states as electrophilic addition transition states. Clearly, there are few structural differences between the four transition state structures for styrene epoxidation. As there are few geometric differences between the various transition state structures, there are also few energetic changes. Therefore, replacement of histidine by N_δ_-methyl histidine in our HRP model appears to have few electronic and structural effects on the first coordination sphere of the metal–heme, the properties of Cpd I and its ability to activate a substrate. Consequently, the mutation of the axial histidine by N_δ_-methyl histidine in HRP has no direct electronic effect on Cpd I, but may affect the structure through perturbations and the breaking of a hydrogen bonding network that affects the overall structure of the enzyme. Thus, the N_δ_-methyl histidine lacks a key hydrogen bonding interaction in the distal pocket and consequently could have a major effect on cytochrome *c* peroxidase, where the radical in Cpd I is not located on the porphyrin ligand but on the distal Trp residue. As such, we decided to investigate C*c*P models and the engineering of their axial ligand to N_δ_-methyl histidine.

**Figure 4:**
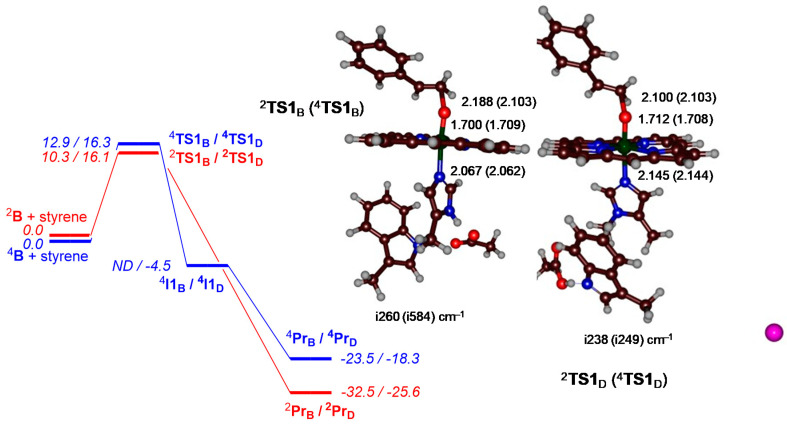
Potential energy landscape of styrene epoxidation by ^2,4^**B** and ^2,4^**D** as calculated at UB3LYP/BS1 in Gaussian-09. Energies (UB3LYP/BS1//UB3LYP/BS2+ZPE+E_solv_) are in kcal mol^−1^ with respect to isolated reactants and give values for the reaction of **B**/**D** and doublet spin results in red and quartet spin results in blue. Optimized geometries of the rate-determining transition states give bond lengths in angstroms and the imaginary frequency in cm^−1^.

To determine whether changes in proximal pocket hydrogen bonding networks due to ligand substitution in C*c*P have an effect on catalysis, we created models of C*c*P and its N_δ_-methyl histidine-ligated mutant (i.e., the HisMe-engineered form), which designated models **B** and **D**. We then tested the styrene epoxidation by these models and the results are displayed in Figure 4. The overall reaction mechanism shown in Figure 4 is similar to that in Figure 3 above and follows a stepwise mechanism leading to epoxides with large exothermicity. The rate-determining barrier for structures **B** and **D** in a reaction with styrene is the C–O bond activation transition state with barriers of 10.3 (12.9) kcal mol^−1^ for ^2^**B** (^4^**B**), while, for model **D**, values of 16.1 (doublet) and 16.3 (quartet) kcal mol^−1^ are found. As such, the replacement of the axial histidine with N_δ_-methyl histidine raises the styrene epoxidation barriers by 3.4–5.8 kcal mol^−1^. This trend is in agreement with experimental work on engineered C*c*P systems that reported a slower reaction with the substrate after engineering. In general, the epoxidation barriers are also higher in energy for models **B**/**D** than those of models **A**/**C** shown above. Previously these differences were explained as originating from the axial ligand hydrogen bonding network that affects the redox potential of Cpd I and raises those in C*c*P [39,73].

Despite the fact that the hydrogen bonding network on the axial site of the heme has been broken in the HisMe models, interestingly, few changes are seen in the optimized geometries of the transition states ^2,4^**TS1**_B_ versus ^2,4^**TS1**_D_. As a matter of fact, they are structurally similar to transition states ^2,4^**TS1**_A_ and ^2,4^**TS1**_C_ (compare Figure 3 and Figure 4). Moreover, for models **B** and **D**, the styrene epoxidation mechanisms do not pass a stable radical intermediate structure. Attempts to identify the radical intermediates on the doublet spin states failed, while ^4^**I1**_D_ is a saddle point and was found to collapse to products rapidly. As such, the lifetimes of these radical intermediates are short in the quartet spin states. 

**Figure 5:**
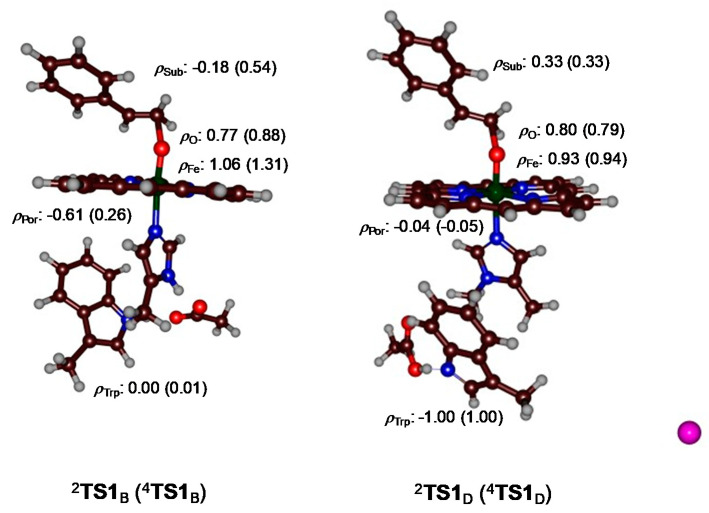
Group spin densities of UB3LYP/BS1 calculated transition states for styrene epoxidation by ^2,4^**B** and ^2,4^**D** as calculated in Gaussian-09.

Although the group spin densities and charges of the pair of doublet spin structures (^2^**TS1**_A_ and ^2^**TS1**_C_) and the pair of quartet spin structures (^4^**TS1**_A_ and ^4^**TS1**_C_) are virtually identical, a different picture emerges for the C*c*P models **B** and **D**. The group spin densities of ^4,2^**TS1**_B_ and ^4,2^**TS1**_D_ are shown in Figure 5 for comparison. Thus, in all four structures, there are about two unpaired spin densities on the FeO unit, although its polarization varies from structure to structure. This unpaired spin contribution of about two originates from the single occupation of the π*_xz_ and π*_yz_ orbitals in all structures that are spread over the FeO group. In the reactant complex ^2,4^**D** there is a third unpaired electron in a dominant π_Trp_ orbital that gives rise to spin density on the Trp group.

In transition state ^2,4^**TS1**_B_, the third unpaired electron appears to be located on the porphyrin macrocycle and a spin of ρ_Por_ = −0.61 (0.26) in the doublet (quartet) spin state is found. At the same time, radical character starts to develop on the styrene group: ρ_Sub_ = −0.18 (0.54) in the doublet (quartet) spin state. By contrast, a full radical is found on the Trp unit in ^4,2^**TS1**_D_ (ρ_Trp_ = −1.00 in ^2^**TS1**_D_ and ρ_Trp_ = 1.00 in ^4^**TS1**_D_). Clearly, the lack of a hydrogen bonding network in the large model has a dramatic effect on the charge distributions and moves the spin density to the Trp unit, while there is none in the hydrogen bonded system.

Since the C–O bond activation transition state (^4,2^**TS1**) results in electron transfer from the substrate into the π_Trp_ orbital, the electron affinity of the Trp group should determine how easily the C–O bond formation can proceed. As the indole group of Trp in model **D** has transferred a proton to the nearby Asp group, the electron affinity is impacted. Indeed, a much higher C–O activation barrier **TS1** is found for complex **D** than for complex **B** (Figure 4).

## 3. Discussion

In this work, we report a series of density functional theory studies on the properties and reactivities of peroxidase models and their N_δ_-methyl histidine engineered mutants. We show that, for small model complexes mimicking HRP, the effects are small and similar structures with the same electronic configuration are found. Moreover, the reaction mechanism and barriers for styrene epoxidation are the same for models **A** and **C**. For the C*c*P model, by contrast, the N_δ_-methyl histidine substitution has a major impact as it disrupts the axial hydrogen bonding network that appears to affect the reduction potential of the Trp radical. As such, the styrene epoxidation barriers go up with the N_δ_-methyl histidine engineered C*c*P in agreement with the experimental observation of a slower reaction.

To understand the differences in reactivity between models **A**, **B**, **C** and **D**, we did a detailed thermochemical analysis of the physicochemical properties of the reactants, as discussed in detail previously [99,100,101]. In particular, we tried to find the differences in electronic properties between **A**, **B**, **C** and **D**, and we calculated the one-electron electron affinities (EA) as defined in Equation (2). For the electron affinity of compound M (EA_M_), we calculated the energy of M and its one-electron reduced form (M^−•^) and took the energy differences in the energies of their optimized geometries, Equation (2). Values calculated at UB3LYP/BS2//UB3LYP/BS1 + ZPE + E_solv_ for structures ^4^**A** and ^4^**C** are 127.5 and 127.2 kcal mol^−1^, respectively. Therefore, the reduction potentials of the HRP model and the N_δ_-methyl histidine-ligated HRP systems are virtually the same. This is in line with the fact that the reaction pathways for styrene epoxidation give similar barriers and structures.

M + e^−^ → M^−•^ + EA_M_ (2)

Next, we calculated the EA for the larger complexes, **B** and **D**. We calculated a value for EA_B_ of ∆E_BS2_ + ZPE = 120.7 kcal mol^−1^ for ^4^**B**, whereas an adiabatic EA_D_ of 115.6 kcal mol^−1^ was calculated for ^4^**D**. Hence, the electron affinity of the N_δ_-methyl histidine engineered C*c*P is slightly lower than that of the wild-type. The experimentally measured reduction potential (E°) can be predicted from the negative value of the electron affinity, see Equation (3) [102,103], and scaled with a constant dependent on the standard hydrogen electrode (SHE) of 4.44 V. Thus, a decrease in EA upon engineering of C*c*P will therefore lead to an increase in the reduction potential. Indeed, experimental work on C*c*P and its N_δ_-methyl histidine engineered form measured an increased redox potential of the engineered C*c*P system [37,38]. Generally, a larger electron affinity corresponds with a faster reaction step with substrates [80,93] and, hence, the lower ^2,4^**D** with respect to **B** should lead to higher substrate epoxidation barriers for **D** compared to ^2,4^**B** based on the difference in electron affinity, which is indeed seen from the landscapes in Figure 3 and Figure 4. Therefore, the loss of the axial ligand hydrogen bonding network weakens the oxidant and makes it more difficult to perform oxygen atom transfer reactions, such as substrate epoxidation.

E° = −EA/23.061 + SHE (4.44 V)(3)

**Figure 6:**
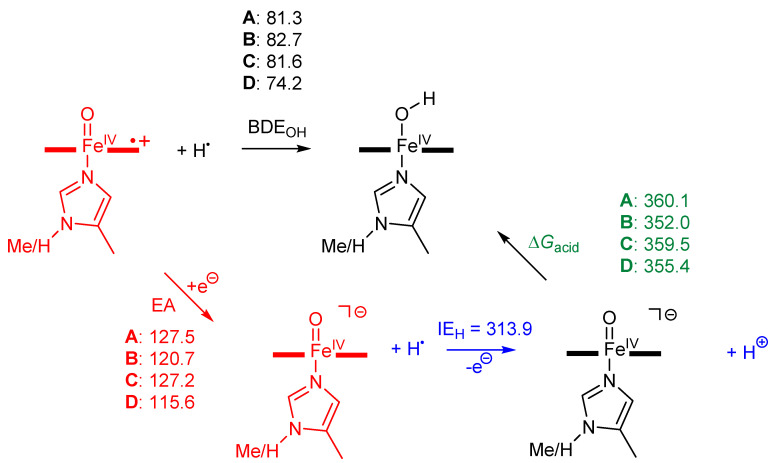
UB3LYP/BS2//UB3LYP/BS1+ZPE+E_solv_ calculated thermochemical properties of reactant complexes **A**, **B**, **C**, **D** with energies in kcal mol^−1^. The bold horizontal bars represent the porphyrin ring.

In addition to the electron affinities of complexes **A**, **B**, **C**, and **D** we calculated several other thermochemical properties including the gas-phase acidity (∆G_acid_) and the energy to form an O–H bond, i.e., the bond dissociation energy of the O–H bond (BDE_OH_). Generally, BDE_OH_ values are useful to understand hydrogen atom abstraction barriers and rate constants [104,105,106,107,108]. Thus, a full thermochemical cycle was calculated (Figure 6) that summarizes these physicochemical properties.

Firstly, the thermochemical cycle includes the one-electron reduction potential (reaction in red on the left-hand side of Figure 6) as the energy difference between Compound I and its one-electron reduced form or the electron affinity (EA) of Compound I. Secondly, the bond dissociation energy of the O–H bond (BDE_OH_) was calculated as the energy difference between the iron(IV)–hydroxo complex and Compound I and an isolated hydrogen atom; see Equation (4). The ionization energy of a hydrogen atom (IE_H_) was taken from experimental data [109]. Finally, the gas-phase acidity of the iron(IV)–hydroxo complex was estimated from the cycle in Figure 6 using the experimental ionization energy (IE_H_) of a hydrogen atom [109], the EA of Compound I and the BDE_OH_ of the iron(IV)–hydroxo complex, as described in Equation (5).

M–H → M^•^ + H^•^ + BDE_OH_ (4)

∆G_acid_ = IE_H_ − BDE_OH_ + EA (5)

The calculated values of EA, BDE_OH_ and ∆G_acid_ values for complexes **A**, **B**, **C** and **D** are given in Figure 6. As discussed above the EA values of **A** and **C** are virtually the same and so are the BDE_OH_ and ∆G_acid_ values. Consequently, replacing an axial histidine ligand with N_δ_-methyl histidine in HRP or analogous peroxidases will have little effect on the electronic configuration of Compound I and its reactivity with substrates. However, in Cpd I species with the radical located on the axial Trp residue, as in C*c*P Cpd I, we see that the EA value drops from 127.5 kcal mol^−1^ for model **A** to 120.7 kcal mol^−1^ for model **B**. On the other hand, the BDE_OH_ values of **A** and **B** are almost the same, so that the difference in gas-phase acidity originates from the differences in the redox potential of **A** versus **B**. Further disruption of the axial hydrogen bonding network through the mutation of the axial histidine by N_δ_-methyl histidine in C*c*P affects the electron affinity and lowers it to 115.6 kcal mol^−1^. Interestingly, despite the fact that the wild-type structures **A** and **C** have quite different electron affinities, actually, their BDE_OH_ values are the same. Moreover, replacing the axial histidine by N_δ_-methyl histidine has little effect on the BDE_OH_ and consequently also not on the ∆G_acid_ values. However, removal of the axial hydrogen bonding network in the C*c*P model, i.e., when comparing structures **B** and **D**, lowers not only the EA value by 5.1 kcal mol^−1^, but also reduces the BDE_OH_ by 8.5 kcal mol^−1^. These two changes, however, cancel each other out with respect to the gas-phase acidity and bring the ∆G_acid_ value of structure **D** close to that of **B**.

## 4. Materials and Methods

Density functional theory (DFT) calculations on active site models of cytochrome *c* peroxidase (C*c*P) and horseradish peroxidase (HRP) enzymes as well as bioengineered proteins were performed using Gaussian-09 [110]. All calculations utilized the unrestricted B3LYP hybrid density functional method [111,112] in conjunction with an LACVP basis set on iron (with core potential) [113] and 6–31G on the rest of the atoms: basis set BS1 [114]. Solvent was mimicked through the continuous polarized continuum model with a dielectric constant representing chlorobenzene, which is close to the value of the actual protein [115]. All structures were geometrically minimized with a solvent model included. To correct the energies, single point calculations were done with a 6–311+G* basis set on all atoms: basis set BS2. These methods and procedures were used previously for analogous systems and reproduced experimental product distributions and rate constants well [116,117,118,119].

## 5. Conclusions

A series of DFT studies on cluster models of peroxidases and engineered peroxidases with an N_δ_-methyl histidine ligand are presented. Surprisingly, the replacement of histidine by N_δ_-methyl histidine has little effect on the structure and reactivity of a minimal active site model containing iron(IV)–oxo and a porphyrin ligand. Even the physicochemical properties, such as electron affinity and gas-phase acidity values, are almost the same. However, the addition of a hydrogen bonding network, as is the case in cytochrome *c* peroxidase, lowers the electron affinity and consequently increases the redox potential, which further affects the gas-phase acidity and BDE_OH_ values. Therefore, the N_δ_-methyl histidine-ligated cytochrome *c* peroxidase should be a weaker oxidant of oxygen atom transfer reactions compared to normal peroxidases and oxygenases.

## Abbreviations

BDE	Bond dissociation energy
C*c*P	Cytochrome *c* peroxidase
HRP	Horseradish peroxidase
ZPE	Zero-point energy
